# Markets, incentives, and health promotion can improve family planning and maternal health practices: a quasi-experimental evaluation of a tech-enabled social franchising and social marketing platform in India

**DOI:** 10.1186/s12889-023-17413-w

**Published:** 2024-01-23

**Authors:** Sumeet R. Patil, Lakshmi Gopalakrishnan, Vishal Sabasu Sai, Richard Matikanya, Payal Rajpal

**Affiliations:** 1 NEERMAN Pvt Ltd, Mumbai, India; 2 University of California San Francisco, San Francisco, USA; 3 Triggerise BV, Amsterdam, The Netherlands; 4 BBC Media Action, New Delhi, India

**Keywords:** Public health, Family planning, Maternal health, Private sector, Market-driven approaches, Social franchising, Social marketing, Community health workers

## Abstract

**Background:**

Improving family planning and maternal health outcomes are critical to achieving the Sustainable Development Goals. While evidence on the effectiveness of government-driven public health programs is extensive, more research is needed on effectiveness of private-sector interventions, especially in low- and middle-income countries. We evaluated the impacts of a commercial social-franchising and social-marketing program – Tiko Platform – which created a local ecosystem of health promoters, healthcare providers, pharmacies, stockists/wholesalers, and lifestyle shops. It provided economic incentives through discounts and reward points to nudge health-seeking behaviors from enrolled women consumers/beneficiaries.

**Methods:**

An ex-post facto evaluation was commissioned, and we employed a quasi-experimental design to compare outcomes related to the use of family planning, and antenatal and postnatal services between users and non-users who had registered for Tiko in three North Indian cities. Between March and April 2021, 1514 married women were surveyed, and outcome indicators were constructed based on recall. Despite statistical approaches to control for confounding, the effect of COVID-19 lockdown on Tiko operations and methodological limitations preclude inferring causality or arguing generalizability.

**Results:**

We found a strong association between the use of the Tiko platform and the current use of temporary modern contraceptives [non-users: 9.5%, effect: +9.4 percentage points (pp), *p*-value < 0.001], consumption of 100 or more iron-folic-acid tablets during pregnancy [non-users: 25.5%, effect: +14 pp, *p*-value < 0.001], receiving four or more antenatal check-ups [non-users: 18.3%, effect: +11.3 pp, *p*-value 0.007], and receiving postnatal check-up within six weeks of birth [non-users: 50.9%, effect: +7.5 pp, *p*-value 0.091]. No associations were found between the use of the Tiko platform and the current use of any type of contraceptive (temporary, permanent, or rudimentary). Effects were pronounced when a community health worker of the National Health Mission also worked as a health promoter for the Tiko Platform.

**Conclusion:**

Commercial interventions that harness market-driven approaches of incentives, social marketing, and social franchising improved family planning and maternal health practices through higher utilization of private market providers while maintaining access to government health services. Findings support a unifying approach to public health without separating government versus private services, but more rigorous and generalizable research is needed.

**Trial registration:**

NCT05725278 at clinicaltrials.gov (retrospective); 13/02/2023.

## Background

Expanding access to family planning and maternal health is essential to achieving the Sustainable Development Goals (SDGs) of reducing maternal mortality to 70 per 100,000 live births, neonatal mortality to 12 deaths per 1,000 live births, and ensuring universal access to sexual and reproductive health-care services. Unintended pregnancies pose an increased risk of maternal and neonatal mortality due to unsafe abortions, lower antenatal care (ANC) utilization, insufficient birth spacing, and high-risk pregnancies [1–6]. A global analysis using Demographic and Health Surveys from 69 low-and-middle-income countries demonstrated that receipt of at least one ANC visit was associated with lower neonatal and infant mortality and malnourishment among children [7]. Another systematic review and meta-analysis of nineteen studies found that the continuum of care for ANC, skilled birth attendance, and postnatal care (PNC) significantly reduced the risk of neonatal, perinatal, and maternal mortality [8]. Consumption of iron folic acid (IFA) during pregnancy can reduce anemia in pregnancy and low birth weight [9].

However, women’s unmet needs for family planning and the gapss in maternal health services are enormous. Globally, 1.1 of the 1.9 billion women of reproductive age (15–49 years) have an unmet need for family planning [10]. More than 50 million women make fewer than four ANC visits, 31 million do not deliver in a facility, and 13 million do not receive needed care for health complications of their infants.

India was the first country in the world to launch a family planning program in 1952 [11]. Since then, the Indian government has launched several programs under the National Health Mission (NHM) [12–14]. These investments in public health have led to improvements in public health indicators. For instance, Maternal Mortality Ratio fell from 600 per 100,000 livebirths in 1990 to 113 per 100,000 live births in 2016–18 [13, 15]. Between 2005–06 and 2019–20, the unmet family planning needs declined from 13.9% to 9.4%. During the same period, women receiving at least four ANC visits increased from 37 to 58%, and institutional births increased from 38.7% to 88.6% [16].

Despite these achievements, reaching the ambitious SDGs for maternal and infant mortality and universal access to family planning will require more significant efforts to address demand- and supply-side barriers to the availability and utilization of these services. Demand-side barriers typically include restrictive social norms related to family planning use, low engagement of male partners, early marriage and early childbearing, shame in obtaining modern contraceptives, lack of women’s agency and decision-making within the household, low educational attainment of women, fear of side effects, and lack of perceived need for ANC prevent women from accessing maternal and family planning services in India [17–20]. The supply-side challenges in India typically include inaccessibility, lack of affordability, poor quality of contraceptives, low information, poor counseling of side-effects of contraceptive methods from providers, and high provider fees [21–23].

There is growing recognition that private sector has to be leveraged to address especially the supply-side barriers [18, 24, 25], considering 80 percent of healthcare in India is provided by the private sector [26]. These private-sector approaches usually include social marketing, high-volume-low cost models, financia incentives, and digital health; see [27] and [28] for a review. Private-sector social franchising models involve a network of self-sustaining small private healthcare businesses that pay a small subscription fee to join the franchisor’s network in exchange for support, including capacity building, branding, social behavior change communication to improve service demand [29, 30]. Social marketing has been used in public health programs by both government and private sector for decades and includes a range of marketing techniques to sell products, services, and promote health practices [31].

While the evidence suggests that social marketing programs can effectively promote behavior change, most of this research is focused on government programs and not the private sector [32]. Additionally, most of the current evidence is focused on issues such as HIV, tuberculosis, and child survival but not on reproductive or maternal health. The limited evidence on the effectiveness of reproductive health social franchises demonstrates increased client volume and satisfaction, but the evidence on healthcare utilization and health impact remain under-studied or mixed, additional research on social franchising within the context of the larger public healthcare system is highly recommended [33]. Impact evaluation of the Tiko Platform seeks to contribute to the thin evidence base and assess the effectiveness of a private sector program that integrates social franchising and marketing concepts on a technology supported platform.

An independent external impact evaluation of Tiko Platform was commissioned by Triggerise (aka Tiko Africa) – a non-profit social enterprise that developed the Tiko Platform and implemented the program in India – with the following objectives: (a) evaluate whether participating in Tiko platform improved access and utilization of family planning and maternal health services for women enrolled in the program (called ‘*Rafikis*’, meaning friend in Swahili); (b) evaluate whether and how Tiko platform benefitted the local health promoters (called pro-agents) economically; (c) develop insights on the merits of the various technical and operational features of Tiko platform; and (d) model population-level impacts of Tiko platform using backend data on service utilization and publicly available health benefits model IMPACT2 developed by Marie Stopes International [34]. This paper is focused on the methods and results only for the first objective. The primary research question that this paper seeks to answer are:

1. Whether the use of the Tiko platform increased utilization by Rafikis of family planning services (specifically, use of contraceptives), and antenatal and postnatal services (specifically, consumption of IFA tablets, ANC check-ups, and PNC check-ups), and
2. Whether and how the above impacts were moderated when community health workers (CHWs) under the National Health Mission (NHM) also worked as health promoters (henceforth, pro-agents) on the Tiko platform.

The findings of this study are relevant to a larger audience interested in family planning and maternal health issues in two ways. First, it adds to a thin evidence base on the effectiveness of private sector interventions in promoting health-seeking behaviors. Currently, a narrow business-focused approach characterizes the private healthcare sector in India, and ‘programmatic’ approaches that can be implemented at scale or seek to create markets are rare. The findings from this study can help build confidence that social-franchising and social-marketing approaches that create local ecosystems similar to the Tiko platform can be one practical approach the private sector can consider. Second, the evidence of increased effectiveness when the pro-agents are also CHWs makes a strong case for integrating public health programs’ reach, scale, and cash benefits with the private sector’s social franchising and incentives-driven health promotion approaches.

## Methods

### Intervention – the Tiko platform in India

**Fig. 1:**
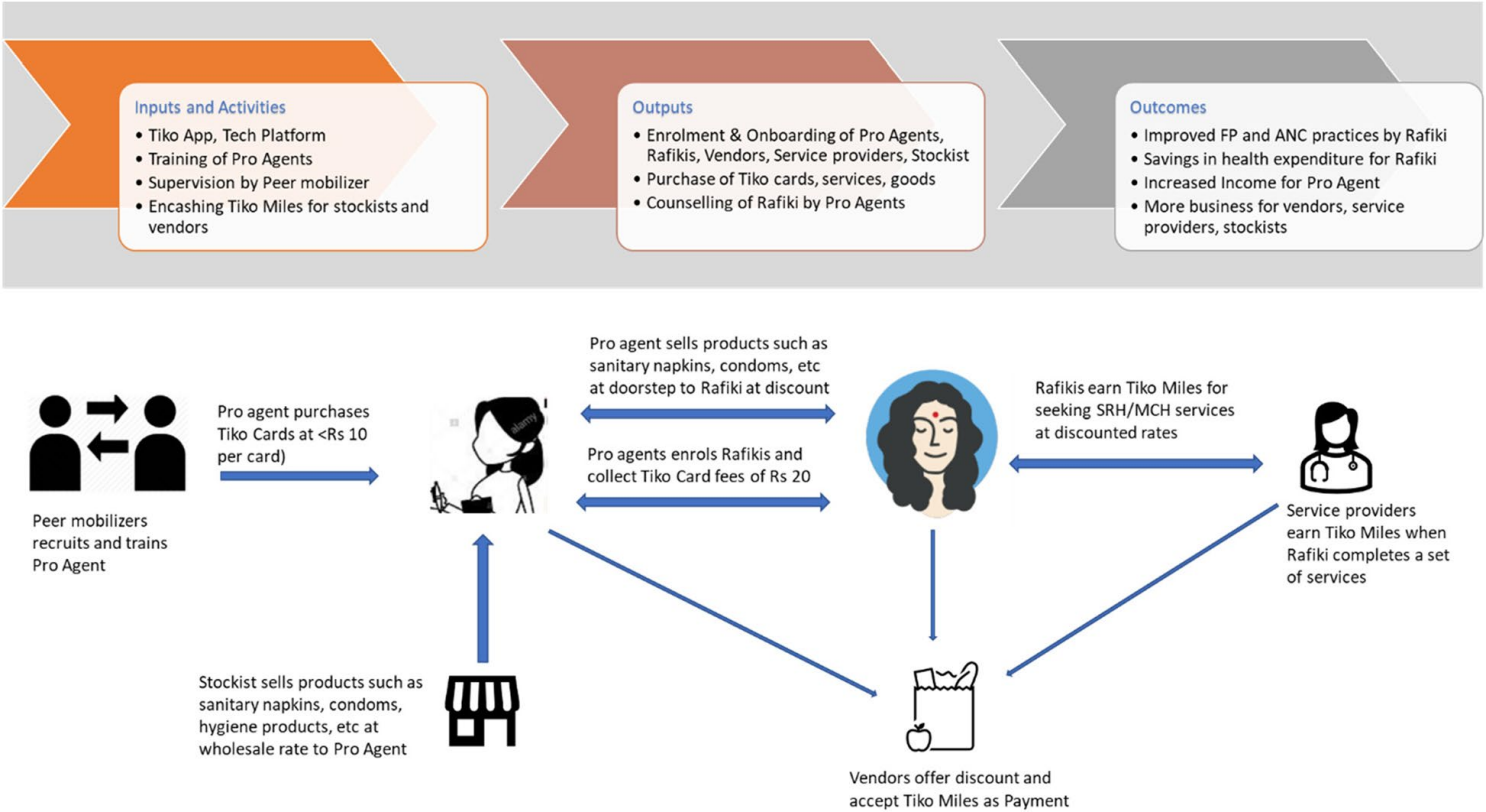
Theory of change for Tiko platform

Figure 1 presents a simplified schematic of the Tiko platform intervention. The main components of the Tiko platform were: (a) a web and phone-based technology that can enroll Rafikis, promote health products/services, track usage, and correspondingly provide incentives in the form of redeemable Tiko miles upon hitting service utilization benchmarks to Rafikis, pro-agents and service providers; (b) pro-agents who onboarded Rafikis on the platform, promoted healthy behaviors, tracked service delivery and utilization, and conducted counseling meetings; and (c) funders who enabled incentives for the Rafikis, pro-agents, and service providers, as well as paid for management and operation of the program.

The Tiko platform leveraged the principles of the social franchising model to create an ecosystem of health service providers and pharmacies (to provide discounted reproductive and maternal health services), lifestyle stores (where the earned Tiko miles could be redeemed by Rafikis, pro-agents and service providers) and stockists (wholesalers form whom interested pro-agents could buy using Tiko miles or cash items such as sanitary napkins, condoms, soaps and other such products, and then self-use or resale these for profit).

The Tiko platform motivated Rafikis to take up services and products using reminders over messages and phone calls, rewards in the form of the Tiko miles on availing services, and follow-ups from pro agents. Rafikis also amass discounts from service providers on the platform, previously negotiated by the Tiko program team as part of the social franchising model ensuring increased business and targeted marketing for service providers on the Tiko platform. Pro agents earned a nominal amount upon onboarding/registering a Rafiki and earned Tiko miles when Rafikis used the services on the platform. Many of the pro agents were also Accredited (Urban) Social Health Activists (referred to as CHWs henceforth) who are health promoters under the NHM and receive performance-based incentives from the government.

Between 2016 and 2019, the Tiko platform had engaged 1,094 Pro-agents, enrolled 54,242 Rafikis, enlisted 62 providers for reproductive and maternal health products and services, and had 52 stockists, retailers, and lifestyle stores and from eight cities in Rajasthan, Uttar Pradesh, and New Delhi.

#### Effect of COVID-19 pandemic on the intervention

**Fig. 2:**
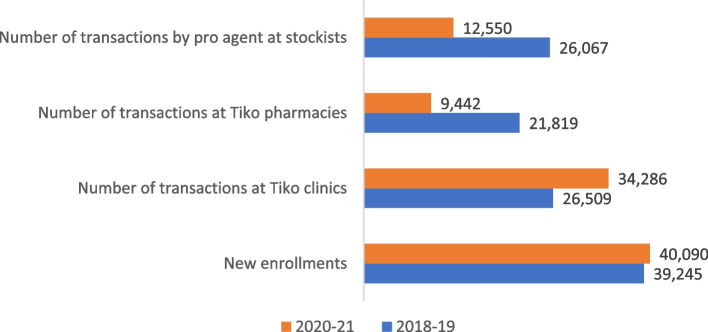
Interactions on the Tiko platform as per backend data captured by Triggerise

Tiko platform started in 2018 in India on a pilot scale and was scaled by mid-2019. However, from January 2020 until June 2021, the COVID-19 pandemic and related restrictions affected how Rafikis interacted with the platform, as shown in Fig. 2. During the lockdown, only medical establishments and pharmacies could stay open and travel for any other purpose, such as a visit to a lifestyle store, was discouraged. Therefore, while the new Rafiki enrolments prior to (2018–19) and during the COVID-19 pandemic (2020–21) were relatively the same, the use of healthcare services was substantially higher during the pandemic, but the redemption of Tiko miles at pharmacies and vendors was substantially lower. The transactions between the pro agents and stockists from whom they could purchase at-discount health goods also substantially declined during the pandemic. CHWs had the added burden of COVID-19-related health promotion, which could have deviated them from their work as pro-agent under the Tiko platform. Market-driven incentives could play a much weaker role during the pandemic because miles could not be redeemed, and pro-agents could not promote health goods. Without Tiko miles being too valuable during the pandemic, Rafikis and healthcare providers also had less incentive to register their visits to earn Tiko miles. However, the providers continued to offer discounts agreed with the Tiko platform when Rafikis showed the Tiko card or App (without registering for the visit event). Therefore, the methods and results need to be assessed and interpreted within the above context of the COVID-19 pandemic.

#### Study sites

Although Triggerise had expanded to eight cities from three states of India, the intervention was at scale and matured in three cities – Jaipur and Ajmer in Rajasthan, and Agra in Uttar Pradesh (UP) – and sampling of both user and non-user Rafikis from the area of operations of a given pro-agent was practicable only in these cities^1^ which included 88 percent of all Tiko users. Therefore, the evaluation study was restricted to these three cities. Table 1 presents key demographic and health statistics for urban population in Jaipur, Ajmer, and Agra districts based on National Family Health Survey (NFHS) 2015–16 [35]. Jaipur and Agra are two of the largest cities in Rajasthan and UP. Both cities have significant differences in several health indicators, including child marriage, unmet need for FP, use of contraceptives, ANC, and institutional births. Ajmer is much smaller than Jaipur and has a similar family planning profile but a worse ANC situation.

**Table 1:** Demographic, Health and Family Planning Statistics in Jaipur, Ajmer and Agra Districts, NFHS 2015-16 Urban Population^a^

ANC and FP related indicator	Jaipur Urban	Ajmer Urban	Agra Urban	India Urban	India Total
Women with 10 or more years of schooling	50.1%	51.1%	48.2%	51.5%	35.7%
Women aged 20–24 years married before age 18 years	22.1%	15.1%	11.7%	17.5%	26.8%
Total unmet need for FP among married women	15.3%	8.9%	8.5%	12.1%	12.9%
Married women currently using any contraceptive method	63.8%	72.0%	63.0%	57.2%	53.5%
Married women currently using any modern contraceptive method	61.1%	64.9%	43.6%	51.3%	47.8%
Mothers who had at least 4 ANC visits (for the last birth in last 5 years)	67.8%	61.5%	56.8%	66.4%	51.2%
Mothers who consumed iron folic acid for 100 days or more when they were pregnant	38.6%	13.2%	22.3%	40.8%	30.3%
Institutional Births	92.7%	90.2%	85.8%	88.7%	78.9%
Institutional Births at Public Facility	53.3%	59.8%	24.0%	46.2%	52.1%

Source: National Family Health Survey (NFHS) 2015–16 [35]

*ANC* antenatal care, *FP* family planning

^a^NFHS sample can include the main city in the district as well as other urban areas so that the presented statistics are not representative of Jaipur, Ajmer, and Agra cities alone

### Impact identification strategy

An *ex-post facto* evaluation was commissioned, and we employed a quasi-experimental design which was the only and most suitable for this evaluation. We compared the outcomes related to family planning and maternal health practices between the Rafikis who availed of the services on the Tiko platform (users) and those who did not access the services (non-users) using a generalized linear model with fixed effects at the pro-agent level. Our ability to estimate causal impacts was severely limited without a baseline measurement and an experimental control group. However, we attempted to control the confounding or selection bias in three ways.

First, we restricted the target population to married women of reproductive age (18 to 50 years) who had registered on the Tiko platform to control for confounders associated with decisions to opt-in to the intervention. However, these restrictions on the target population limit the findings' external validity for the general population of women of reproductive age who may lack access to or who may not opt for a service such as Tiko Platform.

Second, we specified fixed effects at the pro agent level to control for confounders at pro agent level and above. Example of a few confounders include her ability for health promotion, her workload, COVID-19 specific restrictions and work modification, the cultural–geographical-market-social-demographic feature of the area pro agent served, types of service providers and vendors in her area of operations, support provided by Triggerise manager for the city, among others. Therefore, the confounders that needed further control were at the household or Rafiki level.

Third, we present the impact estimates with and without controlling for Rafiki and household level covariates so that differences in the results can suggest the presence of confounding due to these covariates or other confounders correlated with these covariates. It is possible that factors correlated to Rafiki’s decision to ‘use’ the platform also affected the outcomes so that the additive effects of using the Tiko platform over the non-use could be biased. We included Rafikis’ age, education, access to a smartphone and number of children as potential confounders in our model. If the impact estimates changed substantially in magnitude or statistical significance, then we interpreted them as evidence of confounding. However, agreement in the results with or without controlling for these covariates does not necessarily assure causality or attribution.

### Statistical methods

The impact parameter of interest is the marginal treatment effect conditional on sample restriction and enrolment on to the Tiko platform estimated using the following generalized linear model specifications:

1 $${Y}_{ij}= {\beta }_{0}+{\beta }_{1}{T}_{ij}+{M}_{k}+ \varepsilon$$

2 $${Y}_{ij}= {\beta }_{0}+{\beta }_{1}{T}_{ij}+{M}_{k}+{\varvec{\beta}}.{{\varvec{X}}}_{ij}+ \varepsilon$$

where, *Y*_*ij*_ is the outcome of interest for Rafiki *i* from the area served by Pro-agent *j, T*_*ij*_ is a dichotomous indicator for whether or not the Rafiki is an user of the platform or not; M_*k*_ are the fixed effects for *k* pro-agents in our sample; $\varepsilon$ is error term; and ***X***_***ij***_ are a set of covariates such as age, education, access to smart phone and number of children to assess and control for potential confounding due to these characteristics. The coefficient β_*1*_ is the estimate of the impact parameter of interest.

To assess effect modification when the CHW under NHM also worked as a Pro-agent, following models were specified without fixed effects at pro-agent level:

3 $${Y}_{ij}= {\beta }_{0}+{\beta }_{1}{T}_{ij}+{\beta }_{2}{T}_{ij}\cdot {CHW}_{j}+ \varepsilon$$

4 $${Y}_{ij}= {\beta }_{0}+{\beta }_{1}{T}_{ij}+{\beta }_{2}{T}_{ij}\cdot {CHW}_{j}+{\varvec{\beta}}.{{\varvec{X}}}_{ij}+ \varepsilon$$

where, in addition to terms previously explained *CHW*_*j*_ is an indicator variable for pro-agents who work as CHW. The coefficient β_*1*_ is an estimate of the impact of using Tiko platform when pro-agent is *not a* CHW, and (*β*_*1*_ + *β*_*2*_)^β^ is an estimate of the impact of using Tiko platform when pro-agent is also a CHW. In Eq. (4), ***X***_***ij***_ additionally include pro-agent characteristics such as age, marital status, years of education, and caste category to control for potential pro-agent level confounders.

All analyses were done in STATA version 15, documented in a DO file, and replicated by two researchers (VS and SP).

### Primary outcomes and target population

Considering the focus of the Tiko platform on promoting contraceptive use, linking pregnant mothers with doctors on the platform for ANC and PNC, and with pharmacies for IFA, and promoting adherence to the IFA regimen and ANC checkups, five primary outcomes were defined as explained in Table 2. Additional secondary outcomes include the location of receiving ANC or PNC checkups, whether IFA was received free or purchased, and out-of-pocket expenses (OOPE) for these products and services.

**Table 2:** Target population and definition of the indicators for the primary outcomes

Primary Outcome	Target population / Denominator	Numerator
Current prevalence of contraceptive use	Married Rafikis enrolled on the platform	Those who reported to be using any family planning or birth control method whether permanent or temporary at the time of the survey
Current prevalence of modern temporary-contraceptive use		Those who reported to be using, at the time of the survey, temporary and modern contraceptive products such as IUD, injectable, emergency pills, implants, pills, condoms, and diaphragms, but not sterilization, hysterectomy, withdrawal, standard days or lactation amenorrhea methods
Proportion of women who received at least 4 ANC check-ups during their last pregnancy	Married Rafikis enrolled on the platform and those who delivered a child after 1 January 2019 so that the child would have been conceived and delivered during the period when Tiko platform was functional	Those who reported making 4 or more ANC check-up visits to doctors when pregnant with the index child
Proportion of women who consumed at least 100 IFA tablets during their last pregnancy		Those who reported consuming at least 100 doses/tablets of IFA when pregnant with the index child
Proportion of women who received PNC check-up within 6 weeks of the birth of a child		Those who reported receiving health check-up by a doctor for the index child and themselves within 6 weeks of the delivery

### Sampling

The sample for the main study included 1200 Rafikis from the user group and 600 Rafikis from non-user groups, considering multiple research objectives of the larger project described in the background section. However, the sample size for evaluating the impacts on the use of contraceptives was expected to be lower because the target population would include only married women: 500 non-users and 1000 users. This sample was expected to be adequate to detect a relative difference of 0.20 SD from the counterfactual normal distribution ~ N (0,1) with power 0.80, Type I error rate of 0.05, and assuming a design effect of 1.5 due to unequal cluster sizes, unequal distribution of users and non-users, and clustering of Rafikis by Pro-agents [36]. In the case of ANC and PNC-related outcomes, we expected a smaller sample of 200 non-users and 400 users who would have been pregnant and delivered a child during 2018–2021 when the Tiko platform was functional. This sample was expected to be adequate to detect a relative difference of 0.30 SD with similar sampling assumptions as above.

**Fig. 3:**
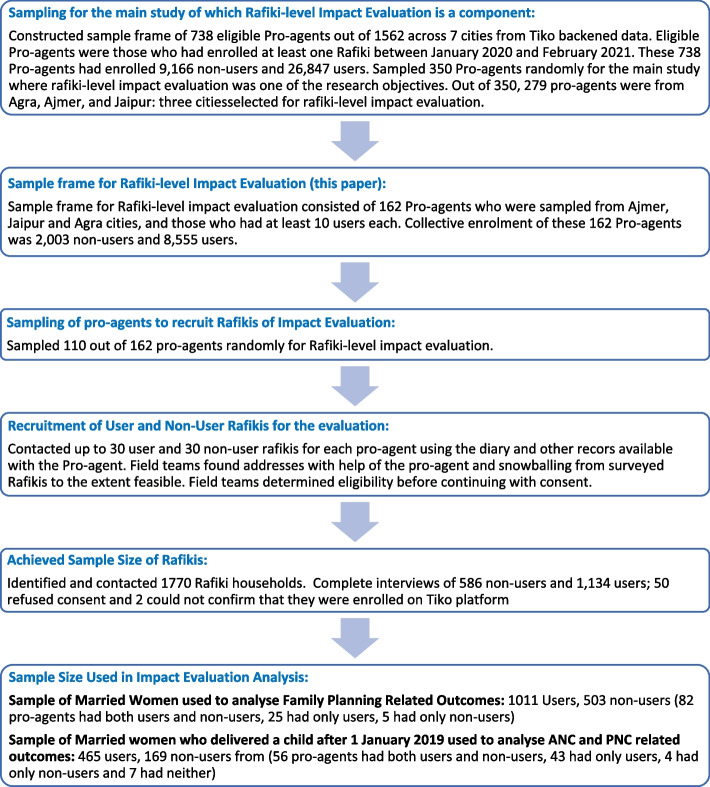
Flow chart for sampling and recruitment of Rafikis

Figure 3 describes how the study participants were sampled in three stages. First, 350 Pro-agents were sampled from a sampling frame of 738 eligible Pro agents from seven cities for the main study, of which this impact evaluation is one component. For the impact evaluation component, 162 pro-agents sampled from the three cities – Agra, Ajmer, and Jaipur – served as the sample frame. Second, 110 pro-agents were selected using simple random sampling without replacement as clusters for the of user and non-user Rafikis’ survey. Third, up to 30 Rafiki users and 30 Rafiki non-users were contacted for a survey for each selected pro-agent. The field team had to rely on purposive methods to sample the Rafikis, such as referring to pro-agents’ diary records and snowballing. However, the same procedures were followed to identify both users and non-users within a given pro-agent’s catchment area. At the time of sampling, it was not known whether Rafikis were married, pregnant, or had delivered a child. Therefore, general questions related to awareness, perceptions, and feedback on the Tiko platform were asked to all sampled Rafikis, In contrast, primary outcomes-related questions were restricted to the relevant target populations.

### Data collection

The survey of Rafikis was conducted between 20 March and 9 April 2022. The interviews were done in Hindi by professional female enumerators using a Computer Assisted Personal Interview (CAPI) App developed on the ODK platform [37]. In compliance with the IRB-approved protocol to reduce the risk of COVID-19, data collection was limited to asking only essential questions from a minimum necessary sample of respondents. Questions related to the primary outcomes, basic socio-economic, demographic, and exposure to the Tiko platform were asked to all 1,718 Rafikis. In contrast, a more extended version which included more detailed questions on socio-economic and demographic details, awareness of the Tiko platform, interactions with pro-agents, and use of Tiko service providers (doctors and pharmacies) and vendors (beauty parlors and grocery stores) were asked to subset of 479 users who were selected randomly by the CAPI App at the time of interviewing.

#### Ethics and approvals

The study was approved by the Institutional Review Board-Keresa Independent Ethics Committee, which is registered with the Central Drugs Standard Control Organization of the Ministry of Health and Family Welfare (India) (IRB Approval Document Number: ECR/308/Indt/KA/2018 dated 06 March 2021). All participants were informed about the study’s aims, their participation was voluntary, and they could withdraw participation at any time without consequences. In addition, signed informed consent was collected from all the study participants on tablets except when respondents refused to sign, and verbal consent had to be administered. The procedures for obtaining informed written or verbal consent to participate in the study were approved by the Keresa Independent Ethics Committee. As a part of the enrolment on Tiko Platform, all Rafikis had permitted to be contacted for promotional and research activities, which enabled NEERMAN to use the backend data for sampling purposes upon signing a non-disclosure agreement with Triggerise. All methods were carried out in accordance with relevant guidelines and regulations.

## Results

Although a richer set of data was collected from Rafikis and pro agents to meet the boarder objectives of the project, we only present the results pertinent to the impact evaluation outcomes related to family planning, ANC, and PNC in this manuscript. Further, we have presented the descriptive statistics limited only to the variables used in the analysis. Also, the terms ‘impacts’ or ‘effects’ mean ‘association’ between the outcomes and use of the Tiko platform.

### Sample characteristics

Table 3 presents characteristics^2^ of user and non-user married Rafikis enrolled on the Tiko platform. The age distribution of users and non-users was similar, but users include slightly higher proportion of Rafikis in 20–25-year age group. Non-user Rafikis tend to have lower education levels and poorer access to smartphones than the users. A higher proportion of users were pregnant or had a child born in the ‘Tiko activity period,’, which we defined as post-1 January 2019, so that the pregnancy period and thus potential for ANC services could start from 1 April 2018 onwards. Overall, the users were those Rafikis who had a slightly higher need for ANC services due to pregnancy or by virtual having a child and also had higher agency in terms of access to smartphones and education levels. These factors may be potential confounders, so we mainly refer to the adjusted model results – Eq. (2) – in discussing the impacts of the Tiko platform.

**Table 3:** Socio-economic, demographic and fertility related characteristics of users and non-users of Tiko Platform

	**Non-Users**	**Users**
**N**	503	1011
**Age of Rafikis**		
18–20	5.8%	5.8%
21–25	40.4%	45.0%
26–30	21.1%	20.9%
30 +	32.8%	28.3%
**Education Level of Rafikis**		
No education	48.7%	40.7%
Primary or lower	20.5%	20.8%
Secondary or lower	21.9%	26.9%
Higher	8.9%	11.7%
**Access to Smartphone for Rafikis**	57.7%	65.4%
**Fertility history related of Rafikis**		
Pregnant at the time of the survey	8.2%	12.2%
Have children	86.7%	87.1%
Whether child was born during after 1 January 2019	33.4%	45.9%

### Exposure to and engagement with the Tiko platform

Nearly all users knew the pro agent, but less than one-third knew their area’s Tiko service providers and vendors. Over three-fourths of the users were aware of the discounts on health services and products at Tiko clinics, and approximately one-third were aware of the discounts at Tiko pharmacies. However, the awareness about earning Tiko miles for using the health services and products – one of the critical components of incentivizing positive health behavior – was just above 12 percent, and only six percent knew that these miles could be redeemed for lifestyle products and services. No meaningful differences existed in the above awareness levels by age, education, or smartphone access levels.

Regarding using the Tiko platform, over half of the users reported receiving three or more visits from the pro-agent. More than 40 percent reported receiving counseling on family planning, maternal health, nutrition, and menstrual hygiene. More than 80 percent of the users (especially the younger ones) availed the services of Tiko platform doctors, and about 50 percent availed of products from Tiko platform pharmacies. More than 30 percent of users reported buying health products (family planning products, sanitary napkins, handwash, etc.) from the pro-agents. However, a small fraction redeemed miles to get lifestyle services and products. As per the discussions with pro-agents and Triggerise field staff, the usage levels could have been affected by the COVID-19-related lockdown, which made access to doctors and pharmacies much easier than lifestyle stores such as a beauty parlor or a grocery shop. Even promotional activities by pro agents had to be limited.

For a subset of users, their perceived benefits of using the Tiko platform compared to the situation before were asked in the context of family planning only. While less than eight percent perceived that the Tiko platform did not benefit them in any way, less than ten percent of the users listed privacy, access, and affordability as benefits. Fourteen percent of the users (slightly higher among younger users) said they were made aware of the importance of family planning and its options. Almost one-fourth (especially among younger users) shifted their family planning service provider to a Tiko platform provider. Few users reported access to more advanced or complex procedures, such as IUDs or abortions, that were made possible on the Tiko platform.

### Impact of the Tiko platform on family planning

In Table 4, we present the additive effects of the use of the Tiko platform on the primary and secondary outcomes related to the use of contraceptives. The effects estimated with and without adjusting for the Rafiki level covariates as per Eqs. (1) and (2) are presented in the table. However, in the discussion, we mainly refer to the adjusted effects given the difference in characteristics of users and non-users, as presented in Table 3.

**Table 4:** Impact of Tiko on family planning-related practices and expenditure

Outcomes	N	Non- Users Mean	Unadjusted Effect	Adjusted Effect
**Primary Outcomes**				
Current contraceptive prevalence rate among married women	1514	26.6%	3.9 pp [*p*-value 0.146]	6.5 pp [*p*-value 0.023]
Modern contraceptive prevalence rate among married women	1514	9.5%	8.0 pp [*p*-value < 0.001]	9.4 pp [*p*-value < 0.001]
**Secondary Outcomes**				
**Current prevalence of specific family planning methods**				
Intrauterine Device (IUD) (modern)	1514	0.6%	1.3 pp [*p*-value 0.123]	1.6 pp [*p*-value 0.110]
Injectables (modern)	1514	0.6%	1.5 pp [*p*-value 0.044]	1.8 pp [*p*-value 0.030]
Pills (modern)	1514	1.0%	2.5 pp [*p*-value 0.025]	3.0 pp [*p*-value 0.017]
Male condoms (modern)	1514	7.2%	1.9 pp [*p*-value 0.284]	2.0 pp [*p*-value 0.307]
Female condoms / diaphragms (modern)	1514	0.0%	0.3 pp [*p*-value 0.462]	0.4 pp [*p*-value 0.323]
Standard Days Method (traditional)	1514	8.5%	-3.3 pp [*p*-value 0.019]	-3.0 pp [*p*-value 0.055]
Withdrawal method (traditional)	1514	4.2%	-2.0 pp [*p*-value 0.077]	-2.0 pp [*p*-value 0.107]
Lactation amenorrhea (traditional)	1514	2.6%	0.6 pp [*p*-value 0.550]	0.3 pp [*p*-value 0.808]
Female sterilization (permanent)	1514	6.0%	-1.5 pp [*p*-value 0.245]	-0.4 pp [*p*-value 0.762]
Hysterectomy (permanent)	1514	1.4%	-0.4 pp [*p*-value 0.488]	-0.1 pp [*p*-value 0.832]
**Money spent on contraceptive methods over the past 24 months by current users (INR)**	449	363	-25.6 [*p*-value 0.873]	7.1 [*p*-value 0.965]

*Abbreviations: pp* percentage-points, *INR* Indian National Rupee

More than one-fourth (26.6%) of the non-users reported using contraceptives or birth control methods. The use of the Tiko platform is associated with a 6.5 percentage point (pp) increase in the current contraceptive prevalence, but the unadjusted effect size is smaller and statistically not significant. Therefore, it is possible that the use of any type of contraceptives, including permanent methods such as sterilization, is not associated with the use of the Tiko platform but is correlated with age, education, and access to smart phones of the Rafikis. However, the effects are consistent and robust in the outcome of using modern and temporary contraceptive methods. Compared to the non-user prevalence of 9.5 percent, the use of the Tiko platform is associated with almost doubling this proportion with a 9.4 pp increase. Considering the current prevalence of specific family planning methods and contraceptives, the increase in the use of modern contraceptives was driven by pills, injectables, and IUDs and associated with a corresponding decrease in the use of traditional methods such as the withdrawal method and standard days method.

One expected benefit of the Tiko platform was the ability to access discounts and use of the Tiko miles, which could reduce out-of-pocket expenditure (OOPE). On average, Rafikis spent INR (Indian Rupee) 363 on contraceptives. However, there was no increase or decrease in this OOPE by use of the Tiko platform, although a higher proportion of users reported using temporary modern contraceptives, suggesting a shift in preference of contraceptives and expenditure pattern but no change in the total expenditure.

As presented in Table 5, the effect of the use of the Tiko platform is strongly and consistently associated with all primary outcomes of receiving four or more ANC checkups (non-users: 18.3%, Adjusted Effect: 11.3 pp, *p*-value 0.007), consumption of at least 100 IFA tablets during pregnancy (non-users: 27.2%, Adjusted Effect: 11.8 pp, *p*-value 0.006), and PNC checkup within six weeks of the child’s birth (non-users: 50.9%, Adjusted Effect: 7.5 pp, *p*-value 0.091).

**Table 5:** Impact of Tiko on antenatal care, delivery and postnatal care practices and expenditure

Outcomes	n	Comparison Mean	Unadjusted Change in the User Group	Adjusted Change in User Group
**Primary Outcomes**				
Received 4 or more ANC check-ups during pregnancy	634	18.3%	11.2 pp [*p*-value 0.007]	11.3 pp [*p*-value 0.007]
Consumed 100 or more IFA does during pregnancy	634	27.2%	11.6 pp [*p*-value 0.006]	11.8 pp [*p*-value 0.006]
Received postnatal check-up in the first 6 weeks of birth	602^$^	50.9%	7.9 pp [*p*-value 0.076]	7.5 pp [*p*-value 0.091]
**Secondary Outcomes**				
Obtained any ANC during Pregnancy	634	97.6%	-0.3 pp [*p*-value 0.883]	0 pp [*p*-value 0.998]
**Location of ANC**				
Tiko doctors	615	0.0%	39.2 pp [*p*-value 0.000]	39.0 pp [*p*-value 0.000]
Other private doctors	615	50.9%	-16.6 pp [*p*-value 0.000]	-17.0 pp [*p*-value 0.000]
Government facility	615	69.1%	-0.4 pp [*p*-value 0.925]	0.1 pp [*p*-value 0.991]
**IFA Receipt and Consumption**				
Received free IFA supply	615	80.0%	4.7 pp [*p*-value 0.231]	4.8 pp [*p*-value 0.217]
Purchased IFA supplements	615	28.5%	13.6 pp [*p*-value 0.003]	13.4 pp [*p*-value 0.004]
**Location of PNC**				
Tiko doctors	342	0.0%	23.9 pp [*p*-value 0.000]	23.6 pp [*p*-value 0.000]
Other private doctors	342	27.7%	11.4 pp [*p*-value 0.111]	12.3 pp [*p*-value 0.090]
Government facility	342	72.3%	-4.5 pp [*p*-value 0.500]	-5.2 pp [*p*-value 0.443]
At Home	342	10.8%	6.3 pp [*p*-value 0.147]	6.1 pp [*p*-value 0.158]
**Expenditure of ANC, delivery and PNC**				
Money spent on ANC check-ups, IFA and nutrient supplements during the pregnancy (INR)	615	3462	-300 [*p*-value 0.454]	-296 [*p*-value 0.462]
Total money spent on delivery and postnatal care (INR)	602	4101	-528 [*p*-value 0.683]	-520 [*p*-value 0.686]

*Abbreviations: pp* percentage-points*, INR* Indian National Rupee*, IFA* Iron Folic Acid*, ANC* Antenatal Care*, PNC* Postnatal Care

The differences in secondary outcomes between users and non-users present more profound insights into the pathways of the above impacts. There are no additive effects of using the Tiko Platform on access to ANC services, given that almost all Rafikis (97.6%) received at least one ANC check-up. However, the location of ANC substantially shifted from other private doctors to Tiko doctors (non-user: 50.9%, adjusted effect: 11.8 pp, *p*-value 0.006), while the use of government facilities for ANC remained unchanged (69.1%). Almost 40 percent of the users reported using Tiko doctors for ANC (by indicator definition, this proportion was 0% among non-users), and a higher proportion of users making four or more ANC visits collectively suggest that this increase has come from a greater number of visits to Tiko doctors.

More than 80 percent of Rafikis reported receiving free IFA supply during their last pregnancy, and the additive effect associated with Tiko use is small and statistically non-significant. However, the proportion of Rafikis purchasing IFA for consumption during the last pregnancy is substantially higher among the users (non-user: 28.5%, adjusted effect: 13.4 pp, *p*-value 0.004), which suggests that users were more motivated and able to consume IFA by ‘investing’ in such purchases. Note, under the NHM, all pregnant women are expected to get a free supply of IFA, but their regularity and supply are not guaranteed and women may not be even aware of such provisions.

Users were more likely to receive at least one postnatal check-up during the first 6-weeks following childbirth. Almost one-fourth of the users (23.6%) received PNC check-ups at a Tiko provider. However, there is also statistically weak evidence of an increase in accessing other private doctors for PNC check-ups (non-user: 27.7%, adjusted effect: 12.3 pp, *p*-value 0.090). The use of government facilities for PNC remained high for both users and non-users.

Finally, Rafikis incurred OOPE on ANC, IFA, and other nutritional supplements (INR 3462), delivery, and PNC (INR 4101). However, using the Tiko platform was not associated with any increase or decrease in these OOPEs, although the proportion of users using these services was much higher, as discussed previously.

### Effect modification with CHW as pro-agent

In Table 6, we present the socio-economic characteristics of pro-agents by those who were also CHWs and those who were not. Out of 110 Pro-agents we had sampled for the Rafiki-level evaluation, 68 also served as CHW and consented to their interview. Out of 42 non-CHW pro-agents, 32 consented to their interview. Compared to non-CHW pro-agents, the CHW pro-agents were older, and a more substantial proportion were married than non-CHW pro-agents. The proportion of pro-agents with a minimum of higher secondary education was more significant among non-CHW pro-agents than CHW pro-agents. Most pro-agents who were also CHWs belonged to backward classes or Scheduled Castes or Tribes. Almost three-fourths of the pro agents of both types purchased health products from stockists at a discount and resold them to earn additional income.

**Table 6:** Characteristics of pro agents

	**Non CHW**	**CHW**	**All**
**N**	32	68	100
**Average Age (years)**	31.1	38.0	35.8
**Education**			
No Education	3.1%	1.5%	2.0%
Primary or Lower Education	12.5%	5.9%	8.0%
Secondary or Lower Education	37.5%	64.7%	56.0%
Higher Education	46.9%	27.9%	34.0%
**Married**	65.6%	91.2%	83.0%
**Caste Category**			
SCST	31.3%	39.7%	37.0%
OBC	21.9%	42.6%	36.0%
Open	46.9%	17.6%	27.0%
**Works as reseller of health products after purchase from stockists**	75.0%	73.5%	74.0%

Table 7 presents the adjusted and unadjusted effect of using the Tiko platform when the pro-agent is a CHW for the five primary outcomes using Eqs. (3) and (4). As previously presented in Table 4, 26.9 percent of non-users were using contraceptives at the time of the survey, but there was no additive effect of using the Tiko platform. However, as presented in Table 7, when the pro agent was a CHW, the use of the Tiko platform was associated strongly with a greater contraceptive prevalence rate (non-users: 26.9%, adjusted effect of Tiko when pro agent is CHW: 11.2 pp, *p*-value 0.000). This effect might have been confounded by Rafiki and pro agent characteristics because the unadjusted effect was much smaller and less statistically significant (non-users: 26.9%, unadjusted effect of Tiko when pro-agent is CHW: 6.7 pp, *p*-value 0.013). The effect of Tiko platform use on the prevalence of modern contraceptive use was much greater and consistent across adjusted and unadjusted models when the pro agent was CHW (non-users: 9.5%, unadjusted effect of Tiko when pro agent is CHW: 12.7 pp, *p*-value 0.000; adjusted effect of Tiko when pro agent is CHW: 15.0 pp, *p*-value 0.000).

**Table 7:** Effect Modification when CHW works as a pro-agent

	**Current Contraceptive Use (any)**	**Current ‘Modern’ Contraceptive Use**	**Consumed 100 or more IFA tablets during last pregnancy**	**Received 4 or more ANC check-ups during last pregnancy**	**Received PNC check-up during the first 6 weeks of birth**
N for Unadjusted and Adjusted Models^a^	Unadjusted: 1514 Adjusted: 1322	Unadjusted: 1514 Adjusted: 1322	Unadjusted: 634 Adjusted: 609	Unadjusted: 634 Adjusted: 609	Unadjusted: 602 Adjusted: 579
Non-User Mean	26.9%	9.5%	27.2%	18.3%	50.9%
Unadjusted Effect with non-CHW Pro-agent	0.5% [*p*-value 0.885]	6.1% [*p*-value 0.016]	0.7% [*p*-value 0.892]	2.2% [*p*-value 0.639]	6.1% [*p*-value 0.296]
Unadjusted Effect with Pro-agent as CHW	6.7% [*p*-value 0.013]	12.7% [*p*-value 0.000]	5.9% [*p*-value 0.175]	6.6% [*p*-value 0.095]	8.9% [*p*-value 0.064]
Adjusted Effect with non-CHW Pro-agent	3.9% [*p*-value 0.282]	5.4% [*p*-value 0.074]	-5.9% [*p*-value 0.296]	-2.6% [*p*-value 0.601]	14.1% [*p*-value 0.025]
Adjusted Effect with Pro-agent as CHW	11.2% [*p*-value 0.000]	15.0% [*p*-value 0.000]	5.9% [*p*-value 0.177]	6.4% [*p*-value 0.098]	8.1% [*p*-value 0.091]

^a^N in adjusted models is lower because of missing data in few of the covariates. Considering data loss is minimal, data imputation was not done for missing observations

Although overall Tiko platform use was strongly associated with consuming 100 or more IFA tablets during the last pregnancy (see Table 5), this effect was not differential by the status of pro-agent as a CHW or not (see Table 7). The association between the use of the Tiko platform and receiving four or more ANC checkups was observed only when the pro agent also played the role of a CHW, and the results are statistically consistent between both models (non-users: 18.3%, unadjusted effect of Tiko when pro agent is CHW: 6.6 pp, *p*-value 0.095; adjusted effect of Tiko when pro agent is CHW: 6.4 pp, *p*-value 0.098). Similarly, the effect of Tiko platform use is observed on PNC checkups when the pro agent was also a CHW (non-users: 50.9%, unadjusted effect of Tiko when pro agent is CHW: 8.9 pp, *p*-value 0.064; adjusted effect of Tiko when pro agent is CHW: 8.1 pp, *p*-value 0.091).

## Discussion

### Limitations of the study

The study suffers from the following methodological limitations, which compromise the internal validity and generalizability of the findings. First, the *ex-post* facto evaluation designed using a quasi-experimental approach can only evaluate associations but not attribution, irrespective of the care in sampling and model estimation to minimize the confounding bias. Second, our measurements are based on self-reports subject to recall bias and social desirability biases. Therefore, differential reporting by users and non-users cannot be ruled out. Third, the results are valid for a specific target population of women from the three study sites who opted to register on the Tiko platform and are not generalizable to other cities or the general population of women of reproductive age. Perhaps only a small percentage of the population would opt for platforms such as Tiko, so the general-population-averaged effect of such intervention would be negligible. However, such private sector interventions are mainly for those who want them because government or other private providers may not be meeting their needs completely. And such a fraction can be small or large depending on the local context. Finally, the intervention was implemented during the COVID-19-related lockdowns. The measurements were conducted right as the lockdown was being lifted, so the findings may not be generalizable to typical periods, as elaborated in the following discussion.

### Interpretation of findings under context of COVID-19

In terms of family planning outcomes, the observed associations are valid during the COVID-19 pandemic but may not be generalizable to non-pandemic periods. For example, fertility preferences shifted towards avoiding pregnancy during COVID-19 times [38, 39]. When measuring ANC and PNC-related outcomes, the indicators capture a mix of the situation before and during COVID-19 lockdown, initiated on 24th March 2020. For example, a Rafiki who conceived in September 2018 and delivered a child in June 2019 would have received ANC, delivery and PNC care in pre-COVID times. On the other hand, a Rafiki who conceived in August 2019 and delivered a child in June 2020 would have received some ANC services in pre-COVID times but delivery and PNC services during the COVID-19 lockdown. Consequently, the findings might differ in non-pandemic normal times.

The lockdown severely curtailed market-based activities, limiting them to essentials, Naturally, for a market-based intervention like the Tiko Platform, this translated to less effective implementation and updating of services as discussed previously. However, clear gaps in implementation were noted in our data. For example, most users were not aware of Tiko miles, an expected element for behavior change. Even among those who aware of Tiko miles, almost no one reported redeeming them, citing reasons such as lack of phone access, misplacement of Tiko cards, and changes in phone numbers. Perhaps, the Tiko platform could have been more effective during normal periods, especially if economic incentives through Tiko miles had been better understood and utilized. However, our data is too limited to explore this aspect further in our analysis.

### Findings in context of past research and evidence

Our findings contribute to the ongoing debate on whether and how social franchising works by presenting evidence of strong associations and arguing the pathways. Past research on social franchise has demonstrated scalability, improved access to people from lower socio-economic backgrounds, higher customer satisfaction, and improved clinic ratings [29, 33]. However, the impacts on family planning practices, especially the current use of modern methods, have been mixed. For instance, Chakraborty and colleagues found that a social franchising intervention focusing on private clinics in urban and rural Kenya was associated with higher use of improved long acting or permanent methods, but had no effect on current use of modern contraceptives [40]. On the other hand, Azmat and colleagues found that a social franchising intervention predominantly in rural pakistan, which also provided vouchers for IUDs, was associated with 22.7 pp increase in the current use of modern contraceptives from the comparison group mean of 26.1% at the endline, but not on sterilization [41].

A study on *Matrika* social franchising program in India is perhaps closer in context to our study because it was implemented in Uttar Pradesh, India, forming network of private and public healthcare providers, conducting social marketing with help of CHWs, and targeted the continuum of care for maternal, new-born, and reproductive health [42]. The study found no association between the intervention and current use of modern contraceptives, receiving at least 3 ANC check-ups, IFA supplementation for 100 days, or postpartum care within 48 hours of delivery. The authors argued the key reasons for the lack of impact were low awareness of the brand and actual utilization of the providers associated with the brand, male healthcare providers when women preferred female providers, use of CHWs in promotional activities (which they were already doing under the NHM and likely to be overburdened with), and no ‘*threat of expulsion*’ or financial incentives for the providers to stay in the programme. Therefore, the locality of the intervention, consumer preferences, and incentives for private markets can moderate the effectiveness of private sector approaches using social franchising and marketing concepts.

The Tiko platform was also associated with doubling the current use of modern contraceptives among users by 9.4 pp, from a 9.5% level among non-users. This effect was driven by increase in the use of products that were promoted by the pro-agents such as pills, condoms, and connecting Rafikis with doctors for IUDs. There was also a decrease in traditional methods, which may result from the counselling by the pro-agents during marketing of the modern methods. CHWs who worked as pro-agents were more effective, but why, given that government programs already incentivized them and arguably CHWs may have been over-burdened during COVID-19 pandemic? We believe this is a case of incentives because under NHM, CHWs get incentives for 2 or 3 years of spacing, permanent methods, and INR 1–2 for home delivery of pills or condoms [43]. However, with Tiko, they could potentially purchase such products at a discount from stockists and sell for a much larger and immediate profit.

Relatively, the proportion of users receiving four or more ANC check-ups was 60 percent more than that for non-users, and the proportion of users consuming 100 or more IFA tablets during pregnancy was relatively 40 percent more than that among non-users. The relative effect of use of Tiko was almost 15 percent on the proportion of Rafikis getting PNC check-ups within six weeks of birth. Considering a high proportion of users continued to use government facilities for these outcomes, and that the impact on these outcomes was stronger when the pro-agent was a CHW, it appears that the Rafikis were able to optimize their use of government services for free services and cash benefits, and Tiko providers for 'some unmet need’ generated because of social marketing. It is possible that these results are driven by the fact that the Tiko platform was based in urban cities where the density of pharmacies and health providers will be high enough to offer quality and access due to market competition, and Tiko could tip the scale in favour of their network through social marketing and discounts.

## Conclusion

While government-led public health programs have yielded rich dividends over time, the role of private sector is argued to be critical to ensure public health for all. In this study, we investigated effectiveness of Tiko platform, a technology-enabled, incentive-based social-franchising, and social-marketing intervention using an *ex-post facto* quasi-experimental evaluation design. Evidence from our study, despite the limitations, strongly associated the use of the Tiko platform with: (a) an increase in uptake of modern methods of family planning; (b) an increase in the consumption of IFA tablets during pregnancy, mainly though increase in purchase of these tablets in addition to their free receipts through government programmes; (c) ensuring a minimum four ANC check-ups by increasing visits to private doctors while continuing to be registered and visiting government health facilities; and (d) ensuring PNC check-ups within six weeks of birth mainly through increased use of Tiko and to a smaller extent through other private doctors while utilization of government facilities also remained high. This study further suggests that CHWs can be more effective in promoting purchase of health products and/or use of private healthcare providers which can be complementary to the services and benefits provided under the national health programs.

Collectively, the evidence suggests that the private sector can meet unmet needs which government services may not fulfill, and both can co-exist, at least in urban areas, to meet the ambitious Sustainable Development Goals related to public health. An integrated health promotion strategy for services and products under both public and private programs can give women a choice to select the level, quality and cost of their services. For example, CHWs can promote health for both government and private programs, and their incentives linked to health outcomes can be detached from the location of the service, whether private or public facility. Private doctors can also be accredited for public health services and integrated with government schemes, or a social franchise can be recognized by the government as a partner. Currently, women who deliver in accredited private hospitals can receive cash benefits under *Janani Suraksha Yojana* scheme of the NHM. However, the public–private partnership in the health sector remains an intense debate with genuine concerns about equity, access and fairness [44]. Any such integrated approach will require deep thinking and planning for inter-operability between public and private healthcare at systems level.

Finally, more evidence on effectiveness of private sector approaches can contribute meaningfully to the debate on role of private sector, provided it is timely and rigorous. Therefore, private sector initiatives, especially those seeking a scale comparable to government health programmes, should invest in a rigorous impact evaluation at the time of conceptualizing such initiatives.

## Data Availability

The datasets generated during the current study are not publicly available because of inclusion proprietary business information and data ownership by Triggerise. However, data that was used for the analysis presented in this manuscript is available from the corresponding author on reasonable request and with permission of Triggerise. The variables and analysis files used for the results presented in this manuscript can be available from the corresponding author on request detailing intended use.

## Abbreviations

ANC: Antenatal Care
CHWs: Community Health Workers
IFA: Iron Folic Acid
INR: Indian National Rupee
NHM: National Health Mission
OOPE: Out of Pocket Expenditure
PNC: Postnatal Care
pp: Percentage Points
SDGs: Sustainable Development Goals

## Footnotes

1 For other research objectives of interest to Triggerise, sampling of Pro agents from all seven cities was done. These objectives or results are not a focus of this manuscript.

2 A longer version of the tool was administered to a subset of users, and it collected richer data on socio-economic characteristics. However, we do not present these results because this data was not captured from non-users, and thus, not used in adjusting impact estimates.
